# Security Management Suitable for Lifecycle of Personal Information in Multi-User IoT Environment

**DOI:** 10.3390/s21227592

**Published:** 2021-11-16

**Authors:** Yong Lee, Goo Yeon Lee

**Affiliations:** 1 Department of Software and Security Convergence, Baewha Women’s University, Seoul 03039, Korea; 2 Department of Computer and Communications Engineering, Kangwon National University, Chuncheon 24341, Korea; leegyeon@kangwon.ac.kr

**Keywords:** Internet of Things, personal information, information lifecycle, IoT suitability, GDPR

## Abstract

In recent years, as all actions of Internet users become information, the importance of personal information is emphasized, but in reality, the management of personal information is still insufficient. With the advent of the concept of sharing systems such as the sharing economy, the numbers of IoT application services (for example, a healthcare service using sharing IoT devices, or a vehicle sharing system with IoT devices) using users’ personal information are increasing, but the risk of using personal information is not managed. To solve this issue, the European GDPR stipulates the content of personal information protection. In this paper, we present a method to securely manage personal information in IoT devices in IoT application environments in accordance with the GDPR. We first describe the lifecycle stages of personal information occurring in IoT application services and propose a method to securely manage personal information at each stage of the lifecycle according to the flow of personal information in IoT devices. We also evaluated the usefulness and applicability of the proposed scheme through two service scenarios. Since the proposed method satisfies the requirements for personal information management in IoT application environments, it is expected to contribute to the development of the IoT business field that handles personal information.

## 1. Introduction

With the advent of mobile handheld devices and various smart devices, the mobility of user devices and Internet application services has become more common, the amount of personal information handled by these services has been increasing, and user terminal features have gradually become compact. Likewise, in the IoT(Internet of Things) environment, IoT devices have become miniaturized so that they can be attached to or wearable on a person’s body and so that they can move with people. As the number of IoT devices capable of such mobility increases and communication capacity increases, the amount of personal information processed by IoT applications is also increasing rapidly. Due to the nature of the IoT environment, there exists a situation in which personal information is processed beyond the existing safe management area, and thus the importance of and interest in personal information protection are growing in the IoT service field [1,2,3,4].

Personal information protection, as indicated in the European General Data Protection Regulation (GDPR), requires the subject of personal information collection to be responsible for compliance and the management of strict procedures throughout the entire process of creation, collection, storage, processing, and destruction in the lifecycle of personal information [5,6,7]. IETF RFC8576 also emphasizes the lifecycle and privacy protection of IoT devices [8]. In many cases, the subject of personal information collection strives to prevent the exposure of personal information by thoroughly managing all devices from the collection to destruction of the information.

**Figure 1:**
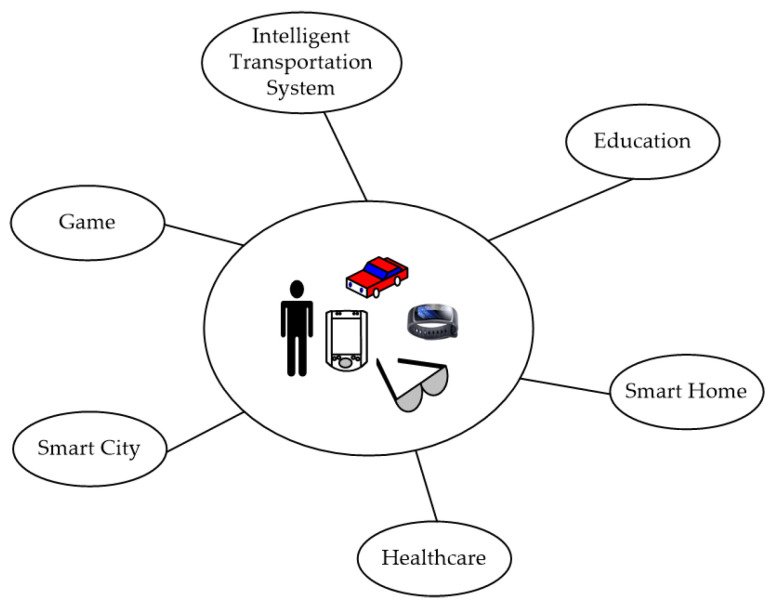
Examples of IoT application using personal information.

In recent years, as businesses using various IoT devices have become active in the IoT environment, many cases in which personal information is stored and processed in IoT devices have developed [4,9,10]. As shown in Figure 1, education, healthcare, smart cities, intelligent transportation system, and games are examples of IoT applications that make up a personal environment. For example, it is possible to store a user-specific setting value of a healthcare device and then reprocess it, or to construct a user’s healthcare environment using the user’s setting value stored in the device.

IoT devices can be installed and operated in various locations depending on the application, and sometimes they are installed beyond a physically secure management space. In this case, an attacker can steal or clone an IoT device, and personal information stored in the IoT device can be exposed to the attacker. Since the business operator is legally responsible for the disclosure of such personal information, it is necessary to prevent this. Even if personal information is encrypted and stored in the IoT device to protect personal information, if the encryption key is also stored in the IoT device, the attacker can obtain the encryption key in the case of theft, extortion, duplication, etc., and decrypt the personal information.

In [8], the authors explained that IoT devices which are not physically protected in the ambient environment could be easily captured by an attacker, and the authors were concerned about the exposure of encryption keys, etc. The authors considered the case in which multiple users interact with the same IoT devices installed in public places and access them in a variety of ways. In this case, it is absolutely important to securely manage the keys that encrypt personal information in the IoT devices. In addition, they said that the personal information of users managed by the IoT device must be securely delivered to the IoT server and should be safely used according to the needs of the application service until it is destroyed.

This paper aims to present a secure management scheme for personal information during the lifecycle, including storage, of personal information in IoT devices in the IoT service environment. In addition, we aim to allow multiple users to manage and access each encryption key and personal information for each user in consideration of the environment in which IoT devices are shared. This paper proposes a method of encrypting and storing personal information in IoT devices and a secure management method of encryption keys, applying them to prevent an attacker from obtaining the stored personal information even if the IoT device is stolen or duplicated. In addition, we design a personal information management mechanism for each lifecycle stage within the IoT device by applying the proposed method and verify its security. This result will contribute to building a system that enables IoT devices to manage personal information for each user and maintain their lifecycle safe in the shared system-based IoT business environment.

The paper is organized as follows. We consider previous work regarding IoT service security and personal information protection in Section 2. We explain the lifecycle of personal information in IoT services and security requirements in Section 3 and consider the IoT service model of the proposed method in Section 4. Section 5 describes the initialization of the IoT device, and Section 6 examines the lifecycle management model of personal information. Section 7 analyses the security requirements. Section 8 concludes the paper.

## 2. Related Work

In this section, we will look at the research on the lifecycle of information and the protection of personal information in the IoT environment.

With the advent of the era of IoT application services and big data, the amount of data in the Internet application field is rapidly increasing, and data management is becoming increasingly complex. Recently, data configuration and management for the entire lifecycle of data used in various fields of the IoT environment has attracted a great deal of attention. Arass et al. [11] defined and analyzed the lifecycle of text to make raw data into smart data in the context of big data, and proposed a smart data lifecycle as a method of modeling the stages of each cycle.

Several studies have dealt with the data processing of IoT systems such as smart cities in urban environments. Hefnawy et al. [12] noted that in order to integrate domain-specific IoT systems into the complete vision of a smart city in an urban environment, the heterogeneity of data sources and the various application domains and numerous interests that span multiple stages of the data lifecycle must be handled. This paper proposed the Service Lifecycle Management concept and Lifecycle Modeling Language to analyze, plan, designate, design, build, and maintain IoT-supported smart city service systems, and the work analyzed them in a demonstration environment of smart parking. Kung [13] outlined the personal information management requirements for smart cities and communities and risk factors in the lifecycle, and explained the relationship between stakeholders.

Sinaeepourfard et al. [14] proposed a data lifecycle model for the purpose of using data effectively throughout the data lifecycle and using it as big data and explained how to efficiently organize large and complex data sets regardless of field and scale. Demchenko et al. [15] introduced the Scientific Data Lifecycle Management model, which reflects the details and key stages of data management in the field of science, and defined requirements for data management, access control, and security. The studies of [12,13,14,15] dealt with the lifecycle of data but do not reflect security vulnerabilities in the IoT environment.

Gruschka [16] discussed GDPR regulations in the context of big data analysis and analyzed various data protection and personal information protection technologies. They also presented and analyzed two research projects as a case study dealing with sensitive data and a measure to comply with data regulation laws. The impact of the proposed technology on the data processing stage and research results according to the types of information that could be a privacy risk and legal requirements was described. Pandit et al. [17] explored the interoperability of information among the various entities mentioned in the GDPR and described various procedures for information flow, including explicit requirements such as structured data or specific data formats. In [16,17], the authors presented a protection method for personal information emphasized by GDPR, but it is not suitable for the IoT device environment.

Alshammari et al. [18] pointed out that handling personal data without an effective data management model can lead to personal information protection violations. In addition, they proposed an abstract personal data lifecycle model to support the management and traceability of personal data to ensure that a system based on a business process complies with privacy requirements.

There are many studies on the security of IoT applications, but most of them are on the communication level, and studies on services are insufficient [1]. IETF RFC8576 describes the lifecycle and privacy protection of IoT devices. Among the privacy protection guidelines, it mentions individual control over the collection and processing of personal information by IoT devices. In addition, it emphasizes the lifecycle transition problem that can take place when devices are sold without properly removing private data, and the inventory attack that can happen when specific information about IoT devices in the possession of a user is disclosed. They cite that research is still underway on a solution for how to deal with these issues in practice. The framework also explains that IoT devices are not physically protected in the surrounding environment and can be easily stolen by attackers, in which case an attacker can attempt to extract private information such as encryption keys or data. They warn that even if data are encrypted, they can be analyzed by attackers during transmission, but the possibility that encrypted information will be exposed along with the key when the IoT device is stolen is overlooked. They also highlight that security issues when multiple users share the same IoT device are very important and complex [8]. In [8], the authors point out that IoT devices in an environment that are not physically protected are vulnerable to attack, emphasize the importance of the lifecycle transition of privacy in IoT devices, and explain that research on this is underway. Our research proposes a solution by designing a protocol for the entire lifecycle transition of privacy for the lifecycle transition emphasized in [8].

Neto et al. [19] argued for the need for a holistic authentication mechanism throughout the lifecycle of IoT devices from manufacturing to disposal, and as a solution to this, proposed Authentication of Things—a protocol that integrates authentication and access control over the entire IoT device’s life.

In [10,20,21], the authors focused on device-to-device communication in a mobile healthcare environment to solve security issues that occur during communication, such as mutual authentication and end-to-end security between devices. They insisted that they have solved the problem by analyzing their proposed method according to security requirements, but they have not been able to solve the security issues for protecting personal information in the IoT devices. In [10,19,20,21], the authors focused on authentication and end-to-end security in communication between IoT devices but did not address the privacy issue.

TLS is a representative protocol that provides authentication, key exchange, integrity, and message confidentiality between two actors. However, it is a heavy protocol to apply to IoT devices in an IoT environment. In addition, there is no protection function for the information stored in the device. Furthermore, it is not suitable to apply when authentication between three actors (device, gateway, server) is required in this IoT environment [22].

Bertino et al. [23] pointed out that with the advent of the IoT, social networks, cloud computing, etc., as personal information protection issues increase in relation to the use of vast amounts of data, it is necessary to adjust the use of data and personal information. They addressed the possibility of attacks on data collection and processing devices in IoT systems and also discussed related concepts and approaches to big data security and privacy. In addition, research topics to be solved on data security and personal information protection in the big data scenario were presented. Torre et al. [24] argued that it can be beneficial to predict undisclosed personal information when collecting data from applications necessary to improve IoT services, but insisted that sharing such data increases the risk of privacy protection. To solve this problem, they proposed a framework for managing privacy issues arising from the unwanted disclosure of personal data. Blobel et al. [25] examined how the GDPR reflects the security and privacy policies of healthcare systems and investigated how to support medical innovation according to GDPR and how this can be implemented based on international standards and specifications.

In [26], a design concept was proposed to develop a GDPR-compliant personal data management platform utilizing blockchain and smart contract technology. The goal of this platform is to provide a decentralized mechanism for both service providers and data owners for the processing of personal data, which does not specifically address the privacy provisions of the GDPR in terms of its safety. Stach et al. [27] pointed out that although explicit consent of the data owner is required when processing personal data for GDPR consideration in smart health services, it is difficult to address many privacy requirements in most IoT privacy systems. In order to solve the problem that users of personal information are overwhelmed by facing too many options or cannot fully participate in the decision process, they proposed EPICUREAN—a recommender-based privacy requirement elicitation approach.

Mustacoglu et al. [28] introduced a method to protect sensitive data using a password-based encryption approach. To encrypt and store sensitive data in the cloud, an encryption key is generated using a password and salt in the registration process, and sensitive data are encrypted using this encryption key. In this system, the password encrypted with the encryption key and the salt value are stored in the database in the cloud. Park et al. [29] analyzed the backup data encryption method of Huawei smartphones. It is described that the hashed value of the user’s password with the MD5 algorithm is used as the data encryption key for the database, and the hashed value of the user’s password with SHA256 is used as the media encryption key in the method used by Huawei.

Since there has not yet been a scheme to manage the lifecycle of personal information in IoT devices deployed in an environment that is not physically protected, this paper presents a method to securely manage the lifecycle of personal information in such an IoT environment.

## 3. Lifecycle of Personal Information in IoT Service

### 3.1. Characteristics of IoT Devices and Lifecycle of Personal Information

#### 3.1.1. Mobility Characteristics of IoT Devices

In IoT applications in which IoT devices have mobility, it is difficult to set up communication with a gateway or an IoT server in advance according to the user’s movement line or the characteristics of the service [4,10]. In this case, even after the personal information collected by the IoT device is delivered to the IoT server, there are cases where the IoT device needs to store and manage the information. Therefore, we need a model that directly manages personal information according to the lifecycle of personal information in IoT devices and a secure protocol that can transfer personal information between the IoT device and the IoT server. When designing such a model or protocol, taking into account the possibility that the IoT device will be stolen or copied, it should be noted that personal information and encryption keys stored in the IoT device may be simultaneously copied.

We look at an example of an IoT application in which an IoT device stores personal information. In home automation applications, IoT devices can store user-specific settings to set customized service environments for each user. As another example, with the advent of the car sharing concept, when multiple users share a vehicle, the driving environment of the vehicle can be set for each driver, and user-specific information can be stored for this purpose. In some cases, the operating environment of the healthcare device may be set for each user, and the use result may be stored so that the healthcare device is used periodically.

#### 3.1.2. Lifecycle of Personal Information

**Figure 2:**
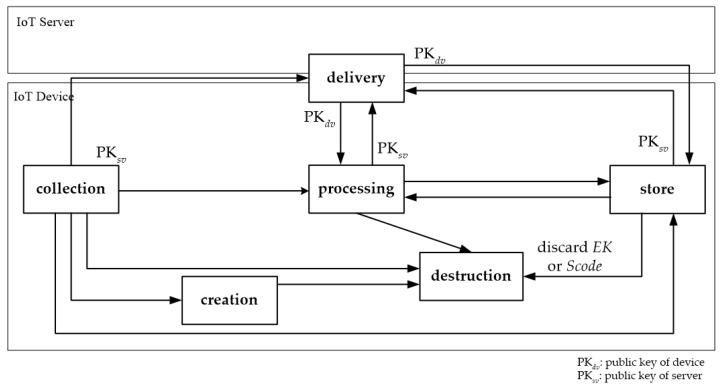
Personal information flow and lifecycle steps.

In this paper, in order to manage the personal information collected by IoT devices in IoT application services, we define a lifecycle of personal information according to the flow of information as shown in Figure 2. This figure shows the lifecycle stage in which personal information is processed within the IoT device and the stage in which it is delivered to the IoT server from the IoT device. First, data about the user’s movement or surrounding environments are created and collected by the IoT device. The collected data are transferred to the IoT server or processed to provide application services. Alternatively, the data may be destructed immediately after they are collected, or they may be stored within the IoT device for use in the next service. The data can be processed or stored in the IoT device and then delivered back to the IoT server or reprocessed. In the flow of information according to the lifecycle process shown in Figure 2, it is necessary to securely protect the personal information of users managed by the IoT device. In particular, it is important to securely process a user’s personal information for each stage of the lifecycle in IoT devices.

### 3.2. Security Requirements for the Lifecycle of Personal Information

In [10,30,31], the security requirements of IoT services or IoT environments are classified in various ways in terms of physical security and service security, communication security, and data security during communication. Since the method proposed in this paper is particularly focused on the security of personal information within IoT devices in the IoT environment, we classify and analyze the following requirements accordingly.

#### 3.2.1. Authentication and Proof of Possession

In order for users to safely use IoT application services, trust between entities participating in IoT applications is required, and an authentication mechanism between them is required [32].

First, in order for an IoT device to collect a user’s personal information, authentication is required between the user and the IoT device. Since many studies have already been conducted on authentication between IoT devices and users, this is not included in this paper. Furthermore, the gateway and IoT server are under the same administration system, so we do not include this topic.

Due to the nature of IoT devices, which may be installed outside of a physically safe management space, in order for the IoT device to collect user information and deliver it to the IoT server, authentication processes are required between the IoT device and the gateway that relays it, and between the IoT device and the IoT server, respectively.

In addition, each entity must be able to prove to the other party that it possesses the private key corresponding to the public key for its own public key pair used in the management protocol.

#### 3.2.2. Nonrepudiation

When delivering personal information between IoT devices and IoT servers, it should be ensured that the sender of the information cannot later deny that the information has been sent. This is provided through the use of a digital signature.

#### 3.2.3. Integrity of Personal Information

To ensure that personal information, such as user IDs or sensing data transmitted between IoT devices and IoT servers, is not forged or altered during transmission, the proposed mechanism must be able to prove the integrity of the information. Thus, it should be possible to prevent IoT service errors or retransmission attacks.

#### 3.2.4. Confidentiality of Personal Information

Personal information managed by IoT applications must be kept confidential while being stored on the IoT device and communicating with the IoT server. Confidentiality must be maintained even when information, such as the user’s ID or IoT device ID, is delivered to the IoT server through the gateway.

In particular, when personal information stored in the IoT device is encrypted and the encryption key is also stored in the IoT device, if the device is stolen or duplicated by an attacker, the attacker can obtain the encryption key and the personal information together. In this case, the attacker will be able to decrypt the encrypted personal information, and the information will be exposed. Therefore, even if the IoT device is attacked, it is essential to prevent the attacker from obtaining the encryption key used to encrypt personal information.

#### 3.2.5. Replay Attack

It should be possible to prevent replay attacks on personal information delivered from IoT devices to IoT servers or from IoT servers to IoT devices.

#### 3.2.6. Access Control of Personal Information and Data Ownership

In the model proposed in this paper, IoT devices can be used by multiple users, and the personal information of multiple users is stored in the IoT device. Therefore, each user must be guaranteed the ownership of their personal information. Furthermore, only the user who is the owner of their personal information should be able to access and use the data.

#### 3.2.7. Characteristics of IoT Services

The personal information lifecycle management protocol proposed in this paper should be able to operate in accordance with the characteristics of IoT devices in the IoT application environment. In addition, it should be able to manage personal information for each user to suit these characteristics.

The characteristics of the IoT application service considered in this paper are as follows. IoT devices can be installed in a physically open place and located outside a secure administrative area. IoT devices can be shared by multiple users and must be able to process each user’s personal information. Therefore, it is necessary to be able to manage the lifecycle of personal information for each user and ensure its security. In order to provide availability, information from other users must be securely available even if one user’s encryption key is attacked. For this, a method for ensuring the security of encryption keys for each user and a method for IoT devices to manage personal information for each user should be presented.

If these requirements are satisfied, the proposed personal information lifecycle management method will have characteristics suitable for providing IoT application services.

## 4. Lifecycle Management Model in IoT devices

### 4.1. Lifecycle Management Model

**Figure 3:**
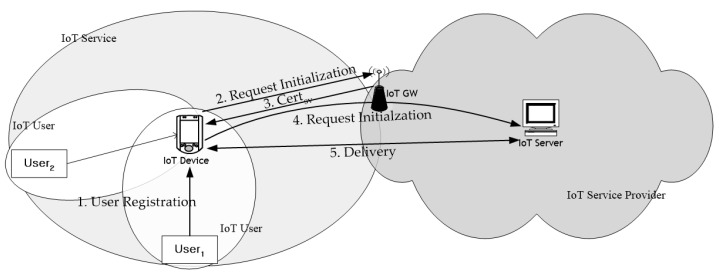
An example of IoT network structure and a simplified procedure.

The IoT application service model consists of IoT devices, gateways, and IoT servers, as shown in Figure 3. In this structure, IoT devices are connected to the IoT server through a gateway, and one IoT device can be used by multiple users. As shown in the figure, the area behind the gateway can be considered safe from attacks because it is within the scope of the IoT service provider’s physical administrative control. The area from IoT devices installed outside this area to the gateway could be the target of attack.

### 4.2. Notations and Assumptions

We make some assumptions for the proposed method.

- Authentication between IoT devices and users has already been dealt with in many studies and is therefore not included in this paper [10,20,21,33]. We assume that the user has registered an identifier and a secret code to the IoT device through a secure channel during the authentication process. A PIN, biometric information, possession-based information, etc. can be used as a secret code. After that, each time the user accesses the IoT device, the user authenticates with an identifier and a secret code. IoT devices store and manage user identifiers and hashed secret codes in a table of IoT devices.
- It is assumed that the gateway and IoT server are physically under the control of the same administration and communicate with each other using a secure channel. The gateway has the IoT server’s certificate and its signature for delivery to the newly registered IoT device.
- It is assumed that various types of IoT devices are used for IoT applications. Considering the weak computing power of IoT devices, it is assumed that a lightweight public key algorithm is used, and cryptographic algorithms are not covered in this paper.

We use the following notations in this paper.

- $ID_{dv}$: IoT device identifier;
- $ID_{usr}$, $Scode_{usr}$: user identifier and secret code;
- $ID_{GW}$: Gateway’s identifier;
- $ID_{sv}$: IoT server’s identifier;
- ts: Time stamp;
- $h(\dot{)}$: One-way hash function;
- ||: Concatenation;
- $Cert_{dv}$: IoT device’s public key certificate;
- $Cert_{GW}$: Gateway’s public key certificate;
- $Cert_{sv}$: IoT server’s public key certificate;
- $PvK_{dv}$, $PuK_{dv}$: Private key and public key pair of IoT device based on public key algorithm;
- $PvK_{GW}$, $PuK_{GW}$: Private key and public key pair of gateway based on public key algorithm;
- $PvK_{sv}$, $PuK_{sv}$: Private key and public key pair of IoT server based on public key algorithm;
- $EK_{usr}$: Encryption key of user;
- $PI_{usr}$: Personal information of user;
- $E({\dot{)}}_{x}$: Encryption with key *x*;
- $D({\dot{)}}_{x}$: Decryption with key *x*;
- $sign({\dot{)}}_{x}$: Signing with key *x* using public key algorithm.

## 5. Initialization of IoT Device

**Figure 4:**
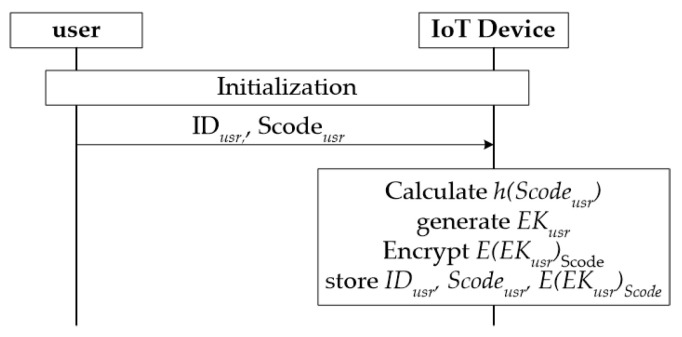
Initialization of user and IoT device.

A IoT device requires an initial setup with a user and initial setup with an IoT server through a gateway. Figure 4 shows these procedures.

### 5.1. User Registration on IoT Device

Except for the authentication between the user and the IoT device, which is not covered in this paper, after the user registers the user ID and secret code on the IoT device, the procedure of the IoT device generating the user’s encryption key and storing it using the secret code is as follows.

1. A user registers $ID_{usr}$ and $Scode_{usr}$ to IoT devices through secure channel;
2. The IoT device receives $ID_{usr}$ and $Scode_{usr}$ from the user and calculates the hash value $h(Scode_{usr})$;
3. The user’s encryption key $EK_{usr}$ is generated;
4. $EK_{usr}$ is encrypted with $Scode_{usr}$ to generate $E{\left(EK_{usr}\right)}_{Scode_{usr}}$;
5. $ID_{usr}$, $h(Scode_{usr})$, and $E{\left(EK_{usr}\right)}_{Scode_{usr}}$ are stored in the user table.

The initial setup between the user and the IoT device is thus completed. Now, to use the IoT application service, the user accesses the IoT device using $ID_{usr}$ and $Scode_{usr}$, and the IoT device authenticates the user by comparing the hash value of $Scode_{usr}$ with the stored $h(Scode_{usr})$. The IoT device can decrypt the user’s personal information after acquiring $EK_{usr}$ by decrypting the stored value $E{\left(EK_{usr}\right)}_{Scode_{usr}}$ of the user’s encryption key with $Scode_{usr}$.

The IoT device does not store the user’s $Scode_{usr}$ and only stores the hash value $h(Scode_{usr})$ in the table, so when the user is not connected, the $Scode_{usr}$ cannot be known and the user’s encryption key $EK_{usr}$ cannot be obtained. Therefore, even if the IoT device is stolen or duplicated by an attacker, the attacker cannot obtain the encryption key because the user’s encryption key is encrypted with the user’s secret code. In addition, since the encryption key for each user is encrypted and managed with each user’s secret code, even if one user’s secret code is exposed, there is a risk that only the user’s encryption key will be known and other users’ personal information can be protected.

### 5.2. User Encryption Key Management in IoT Device

The user’s secret code or encryption key should be periodically updated to enhance security. When updating the secret code or encryption key, the existing secret code or encryption key must be discarded. This process can be performed after obtaining the user’s secret code while the user connects.

Examples of information that an IoT device stores for each user include the user’s ID $ID_{usr}$, $Scode_{usr}$, encryption key $EK_{usr}$, and personal information $PI_{usr}$. Examples of storing a user’s personal information in IoT devices include user environments such as user location during virtual reality games, user-specific settings of medical devices, home automation environments for each user, and driving environments for each user of shared vehicles, which are expected to grow as IoT applications expand. Even fields that are not currently treated as important personal information are likely to be extended to important personal information of users due to the use of big data. Personal information stored on the IoT device is used when the user accesses the IoT device and restarts an application service, reconnects to a game, or restarts a healthcare device. Table 1 shows an example of a table that stores user-specific information in IoT devices.

### 5.3. Initial Setup with Gateway and IoT Server

**Figure 5:**
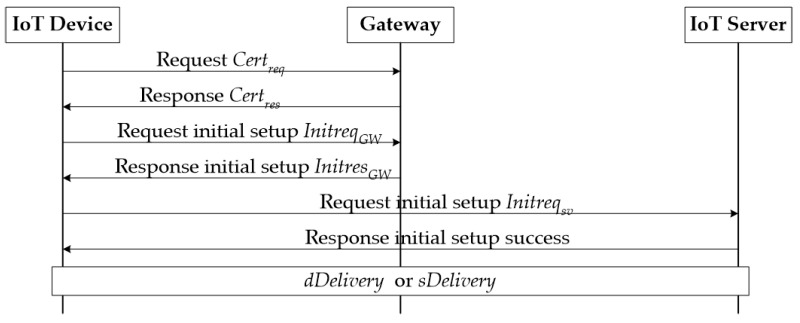
Initial setup between IoT device, gateway, and IoT server.

In order to access the IoT server and use the IoT application service, the IoT device must obtain and authenticate the IoT server’s certificate as shown in Figure 5. An IoT device that aims to communicate with an IoT server through an IoT network first connects to the gateway. The initial setup of an IoT device and a gateway consists of exchanging certificates to authenticate each other, sending an initial setup request, and receiving a response between the IoT device and the gateway. Upon completion of this process, the gateway delivers the certificate of the IoT server to the IoT device, and the IoT device uses it to authenticate and perform an initial setup with the IoT server.

#### 5.3.1. Initialization of IoT Device and Gateway

This section describes the certificate exchange and initial setup procedure between the IoT device and the gateway.

1. The IoT device generates a certificate request message $Cert_{req}$ that signs its certificate $Cert_{dv}$ with its private key $PvK_{dv}$, and sends it to the gateway.
  (1) $$Cert_{req}=Cert_{dv}\left|\right|sign{\left(Cert_{dv}\right)}_{PvK_{dv}};$$
2. Upon receiving the certificate request message $Cert_{req}$, the gateway extracts the public key $PuK_{dv}$ of the IoT device from $Cert_{dv}$, verifies the signature $sign{\left(Cert_{dv}\right)}_{PvK_{dv}}$ included in $Cert_{req}$, and authenticates the IoT device. Then, it generates a certificate response message $Cert_{res}$ that signs its certificate $Cert_{GW}$ with its private key $PvK_{GW}$, and sends it to the IoT device;
  (2) $$Cert_{res}=Cert_{GW}\left|\right|sign{\left(Cert_{GW}\right)}_{PvK_{GW}}.$$
3. After receiving the $Cert_{res}$ transmitted by the gateway, the IoT device extracts the public key $PuK_{GW}$ from $Cert_{GW}$ and verifies the $sign{\left(Cert_{GW}\right)}_{PvK_{GW}}$ contained in $Cert_{res}$ to authenticate the gateway and verify possession of the private key.

An IoT device and gateway that have obtained the other’s certificate perform the following initial setup procedure.

1. The IoT device first concatenates $ID_{dv}$ and ts to generate message $M=ID_{dv}\left|\right|ts$;
2. The IoT device creates M||h(M) by concatenating *M* and its hash value h(M), and generates $sign{\left(M\right)}_{PvK_{dv}}$ by signing it with the private key $PvK_{dv}$;
3. The IoT device combines M||h(M) and $sign{\left(M\right)}_{PvK_{dv}}$ of step 2 and encrypts it with the gateway’s public key $PuK_{GW}$, generating an initial setup request message $initreq_{GW}$ and sending it to the gateway:
  (3) $$initreq_{GW}={E(M||h\left(M\right)||sign\left(M\right)}_{PvK_{dv}}{)}_{PuK_{GW}};$$
4. Upon receiving $initreq_{GW}$, the gateway executes $D{\left(initreq_{GW}\right)}_{PvK_{GW}}$ to decrypt it with private key $PvK_{GW}$;
5. The gateway verifies the IoT device’s signature $sign{\left(M\right)}_{PvK_{dv}}$ included in $initreq_{GW}$ with the IoT device’s public key $PuK_{dv}$ and verifies the possession of the IoT device’s private key;
6. The hash value of *M* is calculated to compare h(M) and verify the integrity of *M*;
7. The gateway extracts the $ID_{dv}$ from *M*, identifies the IoT device, and completes verification for $initreq_{GW}$;
8. Then, to generate the initial setup response message, the gateway concatenates $ID_{GW}$ and ts to generate $N=ID_{GW}\left|\right|ts$;
9. *N*, hash value h(N), the certificate of the IoT server, $Cert_{sv}$ and the signature value $sign{\left(Cert_{sv}\right)}_{PvK_{sv}}$ are combined, and an initial setup response message $initres_{GW}$ is generated by encrypting it with $PuK_{dv}$ and is transmitted to the IoT device.
  (4) $$initres_{GW}=E(N||h\left(N\right)||Cert_{sv}||sign{\left(Cert_{sv}\right)}_{PvK_{sv}}{)}_{PuK_{dv}}.$$

#### 5.3.2. Initialization of IoT Device and IoT Server

After receiving the $initres_{GW}$ from the gateway, the IoT device undergoes a verification process, obtains the IoT server’s certificate, and performs an initial setup procedure with the IoT server.

1. The IoT device performs decryption $D{\left(initres_{GW}\right)}_{PvK_{dv}}$ with $PvK_{dv}$;
2. The IoT device extracts *N* from $initres_{GW}$, finds its hash value, and compares it with h(N) to verify the integrity of *N*;
3. $PuK_{sv}$ is extracted from $Cert_{sv}$ and $sign{\left(Cert_{sv}\right)}_{PvK_{sv}}$ is verified to authenticate the IoT server and verify possession of the IoT server’s private key;
4. $ID_{dv}$ and ts are combined to create message *M*, concatenate *M* and h(M), and generate $sign{\left(M\right)}_{PvK_{dv}}$;
5. The result of step 4 is combined with the certificate of the IoT device, $Cert_{dv}$, and it is encrypted with the public key of the IoT server, $PuK_{sv}$ to generate an initial setup request message $initreq_{sv}$ and send it to the IoT server.
  (5) $$initreq_{sv}={E(M||h\left(M\right)||sign\left(M\right)}_{PvK_{dv}}||Cert_{dv}{)}_{PuK_{sv}};$$
6. Upon receiving the initialization message $initreq_{sv}$ from the IoT device, the IoT server decrypts it with the private key $PvK_{sv}$ and obtains the public key of the IoT device $PuK_{dv}$ from $Cert_{dv}$;
7. The signature value $sign{\left(M\right)}_{PvK_{dv}}$ is verified with $PuK_{dv}$ to authenticate the IoT device and verify possession of the IoT device’s private key;
8. The IoT server extracts *M* from $initreq_{sv}$, calculates its hash value, and compares it with h(M) to verify the integrity of *M*. The IoT server identifies the IoT device by extracting $ID_{dv}$ from *M*;
9. The IoT server delivers the initial setup success message $initres_{sv}$ to the IoT device.

The initial setup process between the IoT device and the IoT server is thus completed, and personal information can be transferred securely.

## 6. Lifecycle Management of Personal Information in IoT Devices

In this section, we consider the management of personal information in IoT devices based on lifecycle stages.

### 6.1. Information Creation and Collection

Recently, as many IoT devices have been developed and commercialized and IoT application systems composed of IoT devices have been built and applied to various fields, various kinds of personal information have been generated and processed, making our lives more prosperous and convenient. In the case of personalized, wearable IoT devices, they are attached to the human body and sense and collect various personal information. There are many types of IoT devices used in personal fields such as games, u-health, smart homes, and education. IoT devices related to the smart city and ITS can also sense and collect personal information due to their service characteristics. Depending on the type of service, the collected personal information is transferred to IoT servers, such as those of healthcare institutions and game servers, and some of them are stored on IoT devices.

The IoT device senses the information and collects it by combining the owner of the personal information $ID_{usr}$, the personal information $PI_{usr}$, and timestamp ts to generate uData.

(6) $$uData=ID_{usr}||PI_{usr}\left|\right|ts.$$

### 6.2. Delivery

The information collected in the previous section can be transmitted to the IoT server. Since personal information may be intercepted during transmission, it must be encrypted with the public key of the IoT server obtained in the initial setup.

Here, we explain the procedure of encrypting and securely transferring personal information between the IoT device and the IoT server.

#### 6.2.1. Delivery from IoT Device to IoT Server

First, we describe the procedure of delivering personal information collected by IoT devices to the IoT server.

1. The IoT device generates message *S* by combining its identifier $ID_{dv}$ and collected personal information uData:
  (7) $$S=ID_{dv}\left|\right|uData;$$
2. The hash value h(S) and the signature value $sign{\left(S\right)}_{PvK_{dv}}$ of *S* are calculated, and then *S*, h(S), and $sign{\left(S\right)}_{PvK_{dv}}$ are combined;
3. The message sDelivery is generated and encrypted with the public key of the IoT server $PuK_{sv}$ and delivered to the IoT server.
  (8) $$sDelivery={E(S||h\left(S\right)||sign\left(S\right)}_{PvK_{dv}}{)}_{PuK_{sv}}.$$

The IoT server receives sDelivery from the IoT device and decrypts it to obtain personal information. The detailed procedure is as follows.

1. The IoT server performs decryption $D{\left(sDelivery\right)}_{PvK_{sv}}$ with private key $PvK_{sv}$ and obtains *S*, h(S), and signature values;
2. Next, $sign{\left(S\right)}_{PvK_{dv}}$ is verified with the IoT device’s public key $PuK_{dv}$ obtained in the initial setup to authenticate the IoT device that is the sender of the message and verify possession of the IoT device’s private key;
3. The hash value of *S* is computed and compared with h(S) to verify the integrity of *S*;
4. The IoT device is identified by extracting $ID_{dv}$ from *S*;
5. uData is extracted from *S* to obtain the user’s $ID_{usr}$ and personal information $PI_{usr}$.

Figure 2 shows that personal information collected, processed, and stored by IoT devices is encrypted and decrypted with the IoT server’s public key pair $PK_{sv}$ when delivered to the IoT server.

#### 6.2.2. Delivery from IoT Server to IoT Device

In this section, we consider how the IoT server encrypts and transmits personal information to IoT devices. This method differs only in that the personal information is encrypted with the public key of the receiving IoT device and is similar to the method by which the IoT device transmits to the IoT server, as described earlier.

1. The IoT server generates $uData=ID_{usr}||PI_{usr}\left|\right|ts$ to send personal information to the IoT device;
2. The IoT server identifier $ID_{sv}$, IoT service-related information $D_{IoT}$, and uData are combined and a *T* message is generated:
  (9) $$T=ID_{sv}\left|\right|D_{IoT}\left|\right|uData;$$
3. The hash value h(T) of *T* and the signature value of *T* with $PvK_{sv}$ are calculated and combined, and encrypted with the public key $PuK_{dv}$ of the IoT device to generate a message dDelivery and deliver it to the IoT device.
  (10) $$dDelivery={E(T||h\left(T\right)||sign\left(T\right)}_{PvK_{sv}}{)}_{PuK_{dv}}.$$

The IoT device receives and decrypts dDelivery from the IoT server to obtain personal information and process or store it as follows.

1. The IoT device decrypts dDelivery using the private key $PvK_{dv}$ and verifies $sign{\left(T\right)}_{PvK_{sv}}$ with the public key of the IoT server to authenticate the sender of the message and verify possession of the private key:
  (11) $$D\left(dDelivery\right)={D(T||h\left(T\right)||sign\left(T\right)}_{PvK_{sv}}{)}_{PvK_{dv}};$$
2. The device extracts *T* from dDelivery to find its hash value and compare it with h(T) to check the integrity of *T*;
3. The device extracts $ID_{sv}$ and $D_{IoT}$ from *T* to identify the IoT server and IoT service type;
4. The IoT device extracts uData from *T* to obtain the user’s $ID_{usr}$ and personal information $PI_{usr}$, and processes or stores it.

Figure 2 shows that the IoT device’s public key pair $PK_{dv}$ is used when the IoT device receives personal information from the IoT server for storage and processing.

### 6.3. Storage of Personal Information

Here, we consider how to directly store personal information collected or processed by the IoT device or received from the IoT server in the IoT device, as shown in Figure 2.

Personal information must be encrypted and stored within the IoT device and must be able to be decrypted as needed. In the proposed method, for this purpose, the IoT device uses a separate encryption key for each user. Encrypting and storing personal information in IoT devices or decrypting and using stored personal information occurs when a user accesses the IoT device. As shown in Table 2, this procedure consists of three steps: decrypting the user encryption key, encrypting or decrypting personal information with the encryption key, and destroying the used encryption key.

1. The IoT device obtains the encryption key $EK_{usr}$ by decrypting $D(E{\left(EK_{usr}\right)}_{Scode_{usr}})$ with the user’s $Scode_{usr}$;
2. The IoT device uses $EK_{usr}$ to encrypt personal information, $(E(PI_{usr}{\left||ts\right)}_{EK_{usr}})$ or decrypt personal information, $D(E(PI_{ust}{\left||ts\right)}_{EK_{usr}})$;
3. The encryption key $EK_{usr}$ and $Scode_{usr}$ should be destroyed when the user terminates the connection or the encryption key is used.

### 6.4. Processing of Personal Information

In order for an IoT device to process personal information, it must directly collect personal information, decrypt the stored information, or receive it from the IoT server as shown in Figure 2.

- When processing personal information created and collected by IoT device, it can be processed without any special pre-processing;
- When receiving and processing encrypted personal information from the IoT server, the IoT device first needs a decryption process in the IoT device according to dDelivery described in Section 6.2.2. The IoT device will then be able to perform appropriate processing on personal information;
- Personal information encrypted and stored in IoT devices can only be processed after decryption by following the procedure described in Section 6.3.

### 6.5. Destruction of Personal Information

Personal information stored in the IoT device should be destroyed when necessary or when the storage period has expired. Alternatively, personal information can be invalidated by destroying the user encryption key stored in the IoT device. In other words, by discarding the encryption key, it is possible to make it impossible to permanently recover the encrypted personal information stored in the IoT device. This can be done by invalidating the user’s $Scode_{usr}$ or by destroying $E{\left(EK_{usr}\right)}_{PW_{usr}}$.

## 7. Security Analysis

### 7.1. Verification of Security Requirements

#### 7.1.1. Authentication and Proof of Possession

In the proposed method, authentication between IoT devices and gateways and between IoT devices and IoT servers is performed using certificates. The IoT device, gateway, and IoT server each possess their own certificates $Cert_{dv}$, $Cert_{GW}$, and $Cert_{sv}$ and transmit them to the other parties to perform the authentication process. The IoT device authenticates the IoT server by receiving and verifying the IoT server’s certificate and its signature. In addition, it is possible to prove possession of the key by signing a message with a private key corresponding to the public key included in the certificate. That is, the message sent by the IoT device to the gateway and the IoT server contains $sign{\left(M||h\left(M\right)\right)}_{PvK_{dv}}$ signed with $PvK_{dv}$ to prove possession of the private key $PvK_{dv}$ corresponding to the public key $PuK_{dv}$.

The gateway and IoT server can verify the signature with the $PuK_{dv}$ contained in the certificate. As a result, it is possible to verify the possession of the key of the IoT device and the authentication of the message sent by the IoT device. The IoT device can verify the signature $sign{\left(Cert_{GW}\right)}_{PvK_{GW}}$ included in $Cert_{res}$ with the gateway’s public key $PuK_{GW}$ and verify possession of the corresponding private key $PvK_{GW}$. In addition, when the IoT device and IoT server exchange personal information, signature verification is performed to verify the authentication of the message. In transferring personal information, the verification of $sign{\left(S||h\left(S\right)\right)}_{PvK_{dv}}$ and $sign{\left(T||h\left(T\right)\right)}_{PvK_{sv}}$ is applied to verify the sender of the message.

#### 7.1.2. Nonrepudiation

Digital signatures are used to provide non-repudiation after personal information is delivered between the IoT device and the IoT server. The IoT device sends $sign{\left(S||h\left(S\right)\right)}_{PvK_{dv}}$ signed with its private key $PvK_{dv}$ to the IoT server, making it undeniable that the message was sent. The IoT server also sends $sign{\left(T||h\left(T\right)\right)}_{PvK_{sv}}$ signed with its private key $PvK_{sv}$ to the IoT device, and the IoT device that verifies this signature has a non-repudiation function.

#### 7.1.3. Verification of Integrity

A timestamp and hash value are used in the proposed method to ensure the integrity of the information delivered between the IoT device and the IoT server. Like $M=ID_{dv}\left|\right|ts$, a timestamp is included to prevent replay attacks, and message $initreq_{GW}$ contains a hash value showing the information the recipient needs to verify integrity. All other messages also contain hash values including timestamps so that integrity can be verified at the receiver.

#### 7.1.4. Confidentiality

In the IoT application environment discussed in this paper, the confidentiality of personal information is required in the following two cases:

- When transferring information between IoT devices and IoT servers, the confidentiality of personal information must be maintained;
- The confidentiality of personal information stored on IoT devices must be maintained.

In each case, we look at how the proposed method guarantees confidentiality. When the user’s ID or the IoT device’s ID is transmitted to the IoT server, it is encrypted with the receiver’s public key to ensure confidentiality. In the $initreq_{GW}$, $initres_{GW}$ and $initreq_{sv}$ messages, the IoT device’s ID is encrypted with the public keys $PuK_{GW}$, $PuK_{dv}$, and $PuK_{sv}$ of the gateway, IoT device, and IoT server, respectively. Since the encrypted information can be decrypted only when each private key, $PvK_{GW}$, $PvK_{dv}$, and $PvK_{sv}$, corresponding to the public key is in possession, the confidentiality of the IoT device’s ID can be guaranteed during transmission. In messages sDelivery and dDelivery, the user’s $ID_{usr}$ and personal information $PI_{usr}$ are encrypted with the public key $PuK_{sv}$ of the IoT server and the public key $PuK_{dv}$ of the IoT device, respectively, to ensure the confidentiality of the personal information when transmitted.

Secondly, personal information stored in the IoT device is encrypted with the user’s encryption key $EK_{usr}$ and stored in the form of $E(PI_{usr}{\left||ts\right)}_{EK_{usr}}$. The encryption key $EK_{usr}$ is encrypted with the user’s $Scode_{usr}$ and stored in the form of $E{\left(EK_{usr}\right)}_{Scode_{usr}}$. The IoT device does not store $Scode_{usr}$, but only its hash value $h(Scode_{usr})$, so if the user does not connect, $Scode_{usr}$ required to decrypt $E{\left(EK_{usr}\right)}_{Scode_{usr}}$ cannot be obtained, and consequently $E(PI_{usr}{\left||ts\right)}_{EK_{usr}}$ cannot be decrypted.

Therefore, even if an IoT device is stolen or duplicated by an attacker, it is possible to guarantee the confidentiality of personal information $PI_{usr}$ because it is impossible to obtain an encryption key to decrypt personal information.

#### 7.1.5. Replay Attack

A timestamp is used to prevent replay attacks of personal information delivered between the IoT device and the IoT server. As in $M=ID_{dv}\left|\right|ts$ and $uData=ID_{usr}||PI_{usr}\left|\right|ts$, messages contain timestamps to prevent replay attacks. In all other messages, a timestamp is included in the hash value to prevent replay attacks.

#### 7.1.6. Access Control and Ownership of Personal Information

In the proposed method, when personal information is stored in an IoT device, it is encrypted and stored with a user-specific private key. The encryption key for each user is encrypted with the user’s Scode, which only the user can know. Therefore, personal information stored in IoT devices can only be accessed by users with an Scode. In addition, it is possible to confirm that the user who has the encryption key capable of decrypting the encrypted and stored personal information is the owner of this personal information.

#### 7.1.7. Suitability for IoT Environment and Availability

The characteristics of the IoT application environment considered in this paper and its requirements are as follows.

- Since IoT devices can be installed in physically open places or beyond safe administrative areas, it must be possible to protect personal information from risks such as theft or duplication of IoT devices;
- Since IoT devices can be accessed by many users and shared by multiple users, it must be possible to separately process and manage personal information and encryption keys for each user;
- The IoT device should be able to manage personal information at each stage of its lifecycle.

In the case of the first requirement, in the proposed method, personal information is encrypted with an encryption key and stored, and the encryption key is encrypted with the user’s secret code and can be obtained only when the user attempts access. Therefore, even if an attacker steals or duplicates an IoT device through a physical attack, the user’s personal information and encryption key cannot be obtained.

Second, it is possible to separately manage personal information and the encryption key for each user. Personal information for each user is encrypted with an encryption key for each user, and the encryption key is also encrypted with a secret code for each user and stored. Thus, even if one user’s information is exposed, the other user’s information can be kept safe and remain available.

Finally, as shown in Section 6, the proposed method is suitable for IoT environments by presenting a procedure to securely manage personal information at each stage of the lifecycle such as creation, collection, processing, delivery, storage, and destruction in IoT devices.

Table 3 summarizes the functions that satisfy each of the security requirements verified above.

Table 4 categorizes security requirements for delivery, storage, and destruction during the lifecycle phase of personal information.

### 7.2. Case Study

To evaluate the method proposed in this paper, we consider a scenario in which a healthcare service using an IoT device operates as follows. Alice’s home has a 3D headset shared by her family that is used for the healthcare service. This 3D headset manages information such as blood pressure, pulse rate, weight, frequency of exercise, exercise cycle, oxygen saturation, and brain waves as follows:

$$\begin{array}{c} PI_{Alice}=\{blood\;pressure,pulse\;rate,weight,frequency,cycle,oxygen\;saturation,\\ brain\;waves\}. \end{array}$$

It also sends the user’s information to the server of the healthcare center and receives the necessary information for the appropriate healthcare from the server. Each family member manages their healthcare information in the 3D headset with their ID and Scode, and the 3D headset encrypts and stores the healthcare information of family members using their respective Scode and encryption key. One day, Alice logs in to the 3D headset with her ID, $ID_{Alice}$, and Scode, $Scode_{Alice}$, and starts the healthcare service. The 3D headset decrypts Alice’s encryption key $EK_{Alice}$ stored in the device using Alice’s Scode $Scode_{Alice}$ and decrypts Alice’s healthcare information $PI_{Alice}$ using this key $EK_{Alice}$. Alice then begins to use the healthcare services using this information. After a while, a courier delivery comes to Alice. Alice takes off the 3D headset, pauses it, and goes to receive the package. At this time, the 3D headset encrypts Alice’s information $PI_{Alice}$ with Alice’s encryption key $EK_{Alice}$, stores it, $E{\left(PI_{Alice}\right)}_{EK_{Alice}}$, and deletes the encryption key $EK_{Alice}$. In the meantime, Alice’s brother Bob logs in with his ID, $ID_{Bob}$, and Scode, $Scode_{Bob}$, and searches Alice’s information to find out her weight and healthcare information. However, Alice’s information is encrypted and stored with Alice’s encryption key, so Bob cannot access it. Alice, who returned, uses her own Scode, $Scode_{Alice}$, to reload her information $PI_{Alice}$ and continues to use the healthcare service. When Alice logs out after completing the healthcare service, the 3D headset adds a timestamp to Alice’s information $uData=ID_{Alice}||PI_{Alice}\left|\right|ts$, hashes it, signs it with its own private key, encrypts it, generates sDlivery in Equation (8), and sends it to the healthcare server. The healthcare server receives and decrypts Alice’s information $uData=ID_{Alice}||PI_{Alice}\left|\right|ts$, adjusts Alice’s healthcare plan, and sends it on the next connection to Alice.

Another scenario can be considered. Charlie signed up for a vehicle sharing system to carry out a crime, and one day rented a car and drove all night. The police were aware of Charlie’s criminal activities in advance and tried to tap the GPS information that the car delivered to the vehicle sharing server while driving. However, the information transmitted from the car to the vehicle sharing management server was encrypted as in Equation (8), so the police could not interpret it. Charlie returned the car at dawn the next day. Charlie’s GPS records are encrypted with Charlie’s encryption key and stored in the shared vehicle. The car also has GPS records of many other users who rented the car encrypted with their respective encryption keys. The police then received a search warrant to investigate Charlie’s car and examined Charlie’s GPS records in the car. At this time, the police can only obtain Charlie’s Scode, so they cannot access other users’ GPS information.

## 8. Conclusions

As the importance of personal information protection is emphasized, in compliance with the personal information protection laws such as the European GDPR, the subject of collecting personal information must comply with and manage strict procedures for each lifecycle of personal information such as creation, collection, storage, delivery, processing and destruction. Because IoT devices can be installed and operated outside of a physically safe management area due to the nature of IoT application services, it is necessary to prevent the exposure of personal information with a secure management method directly from the IoT device for the flow of personal information from collection to destruction.

Existing research focuses on authentication and end-to-end communication security between IoT devices and servers, as well as the physical security of IoT devices. Although IoT devices deal with important personal information, research on the lifecycle management model of personal information in addition to the confidentiality of personal information within IoT devices is still insufficient. In addition, as stated in IETF RFC 8576, the increasing number of IoT devices shared by multiple users will further complicate the management of the lifecycle of personal information within IoT devices. In this paper, we focused on these two problems and solved them.

In this paper, in order to prevent the exposure of personal information due to theft, duplication, eavesdropping, etc. by an attacker for IoT devices, we proposed a management procedure for each stage of the lifecycle of personal information. In the proposed method, personal information is encrypted using a user-specific encryption key, and the encryption key is encrypted with each user’s secret code so that an attacker cannot obtain personal information even if the IoT device is stolen or copied. The user’s secret code will be able to expand variously into biometric information and possession-based information in the future. Only encrypted personal information is stored in the IoT device, and when personal information is exchanged with the IoT server, it is encrypted and transmitted to prevent the exposure of personal information. In addition, it is optimized for an IoT environment that various users access by suggesting a method using encryption keys for each user.

The proposed method enables IoT devices to satisfy the personal information management requirements strictly required by personal information protection laws such as GDPR and is therefore expected to contribute significantly to the development of the IoT business field that handles personal information. In addition, it is believed that the proposed method can be used in various ways in IoT application environments where personal information must be securely managed for each user.

Taking into account the situation in which multiple users share IoT devices, this paper proposed a personal information lifecycle management model, but we have yet to consider the scalability of our approach. Therefore, it is necessary for future research to consider the expandability of IoT services and users who enable the operation of the shared system using the mobile IoT system.

## Figures and Tables

**Table 1:** An example of a table that stores user information in IoT devices.

User ID	User Scode	User Encryption Key	Personal Information
$ID_{usr}$	$h(Scode_{usr})||ts$	$E{\left(EK_{usr}\right)}_{Scode_{usr}}\left|\right|ts$	$E(PI_{usr}{\left||ts\right)}_{EK_{usr}}$
⋮	⋮	⋮	⋮

**Table 2:** Storage procedure of personal information on IoT devices.

Get $Scode_{usr}$
Decrypt $D(E{\left(EK_{usr}\right)}_{Scode_{usr}})$
Encrypt $(E(PI_{usr}{\left||ts\right)}_{EK_{usr}})$ or Decrypt $(D(E(PI_{ust}{\left||ts\right)}_{EK_{usr}}))$
Destroy $EK_{usr}$ and $Scode_{usr}$

**Table 3:** Satisfaction of security requirements by the proposed method.

Security Requirements	Satisfaction	Functions
Authentication	Yes	$sign{\left(Cert_{dv}\right)}_{PvK_{dv}}$, $sign{\left(Cert_{GW}\right)}_{PvK_{GW}}$, $sign{\left(Cert_{sv}\right)}_{PvK_{sv}}$
Message authentication	Yes	$sign{\left(S||h\left(S\right)\right)}_{PvK_{dv}}$, $sign{\left(T||h\left(T\right)\right)}_{PvK_{sv}}$
Nonrepudiation	Yes	$sign{\left(S||h\left(S\right)\right)}_{PvK_{dv}}$, $sign{\left(T||h\left(T\right)\right)}_{PvK_{sv}}$
Integrity	Yes	h(S), h(T)
Confidentiality	Yes	${E(S||h\left(S\right)||sign(S||h\left(S\right))}_{PvK_{dv}}{)}_{PuK_{sv}}$,
		${E(T||h\left(T\right)||sign(T||h\left(T\right))}_{PvK_{sv}}{)}_{PuK_{dv}}$,
		$E{\left(EK_{usr}\right)}_{Scode_{usr}}$, $E(PI_{usr}{\left||ts\right)}_{EK_{usr}}$
Replay attack	Yes	$ID_{dv}\left|\right|ts$, $ID_{GW}\left|\right|ts$, $ID_{usr}||PI_{usr}\left|\right|ts$
Access control	Yes	$Scode_{usr}$ and $EK_{usr}$
Personal Information ownership	Yes	$Scode_{usr}$ and $EK_{usr}$
Availability	Yes	$E(PI_{usr}{\left||ts\right)}_{EK_{usr}}$ and $E{\left(EK_{usr}\right)}_{Scode_{usr}}$

**Table 4:** Security properties required in the lifecycle stages of personal information.

Lifecycle Stage	Security Properties
	Message authentication
	Nonrepudiation
Delivery	Integrity
	Confidentiality
	Replay attack
	Access control of personal information
Store	Personal information ownership
	Confidentiality
	Availability
Destruction	Personal information ownership
	Access control of personal information
